# Biological Considerations in Scaling Up Therapeutic Cell Manufacturing

**DOI:** 10.3389/fphar.2020.00654

**Published:** 2020-05-13

**Authors:** Darshana S. Cherian, Tejasvini Bhuvan, Laurence Meagher, Tracy S. P. Heng

**Affiliations:** ^1^Department of Anatomy and Developmental Biology, Biomedicine Discovery Institute, Monash University, Clayton, VIC, Australia; ^2^Department of Materials Science and Engineering, Monash University, Clayton, VIC, Australia

**Keywords:** mesenchymal stromal cells, immunomodulatory, secretome, cell therapy, biomanufacturing, bioreactors, microcarriers

## Abstract

Cell therapeutics — using cells as living drugs — have made advances in many areas of medicine. One of the most clinically studied cell-based therapy products is mesenchymal stromal cells (MSCs), which have shown promising results in promoting tissue regeneration and modulating inflammation. However, MSC therapy requires large numbers of cells, the generation of which is not feasible *via* conventional planar tissue culture methods. Scale-up manufacturing methods (e.g., propagation on microcarriers in stirred-tank bioreactors), however, are not specifically tailored for MSC expansion. These processes may, in principle, alter the cell secretome, a vital component underlying the immunosuppressive properties and clinical effectiveness of MSCs. This review outlines our current understanding of MSC properties and immunomodulatory function, expansion in commercial manufacturing systems, and gaps in our knowledge that need to be addressed for effective up-scaling commercialization of MSC therapy.

## Introduction

Mesenchymal stromal cells (also known as mesenchymal stem cells; MSCs) are fibroblastic precursor cells isolated in the stromal fraction of many adult tissues, including bone marrow, adipose tissue, and umbilical cord (Musiał-Wysocka et al., 2019). Originally described as bone marrow stroma-derived stem cells capable of forming ectopic hematopoietic niches (Owen and Friedenstein, 1988), MSCs were used in clinical trials for skeletal tissue repair (Owston et al., 2016). Aside from skeletal repair, it is now well recognized that MSCs have many more potential therapeutic benefits due to their immunomodulatory effects on innate and adaptive immune cells. These effects have been largely attributed to their secretory products, including immunoregulatory cytokines and molecules, growth factors, and exosomes (Castro et al., 2019). Animal and other preclinical studies have shown MSCs to be highly efficacious in wide range of ischemic, degenerative, metabolic, inflammatory, or autoimmune disease conditions (Galipeau and Sensebe, 2018; Martin et al., 2019), fuelling enthusiasm for their clinical translation. However, the large quantities of MSCs required for clinical application necessitate generation by larger scale manufacturing processes, including microcarrier-based systems in bioreactors. It is not well understood how these manufacturing processes may alter the MSC immunophenotype and secretome, compared to smaller scale, more conventional planar culture, potentially impeding therapeutic application (Vizoso et al., 2017). In this review, we detail cell manufacturing technologies used currently for MSC expansion and examine the knowledge gap in how such processes may impact on the biological properties and function of MSCs.

## Mesenchymal Stromal Cells

As defined by the International Society for Cellular Therapy (Dominici et al., 2006), MSCs are plastic-adherent when cultured in tissue flasks under standard conditions, express CD73, CD90, and CD105, but lack CD45, CD34, CD14/CD11b, CD79α/CD19, and HLA-DR, and can differentiate into osteoblasts, adipocytes, and chondroblasts under standard differentiating conditions (Ullah et al., 2015). As this set of minimal criteria does not require clonal analyses or stringent *in vivo* studies, the MSCs used in different studies display significant batch-to-batch variations in phenotype and function (Wilson et al., 2019).

### Tissue Sources of MSCs

Early MSC research focused on bone marrow-derived MSCs (BM-MSCs). However, bone marrow aspiration is highly invasive, painful, and increases the likelihood of donor-site morbidity (Strioga et al., 2012). MSCs have since been isolated from almost all postnatal tissues (da Silva Meirelles et al., 2006), including umbilical cord (Bieback and Kluter, 2007), placenta (Wu et al., 2018), dental pulp (Gronthos et al., 2000), and adipose tissue (Zuk et al., 2001). Of these tissue sources, adipose-derived MSCs (A-MSCs) are the most commonly investigated alternative to BM-MSCs. The approach of obtaining MSCs from subcutaneous adipose tissue obtained *via* lipectomy or liposuction has several advantages. The procedures involved are well established, conducted under local anesthesia, relatively non-invasive, and carry minimal risk and discomfort (Zuk, 2013). Excess adipose tissue, frequently discarded as medical waste, provides a valuable source of MSCs which are at approximately 500 times the concentration of BM-MSCs in bone marrow (Fraser et al., 2006; Hass et al., 2011). While BM-MSCs display increased osteoblast and chondroblast differentiation potential, A-MSCs have greater proliferative and secretory capacity (Li et al., 2015). Several studies have reported that A-MSCs exhibit greater immunomodulatory potential (Melief et al., 2013b; Menard et al., 2013), mainly due to increased production of a key molecule involved in T cell suppression, indoleamine-2,3-dioxygenase (IDO) (Menard et al., 2013). Whether these differences translate to increased therapeutic efficacy in clinical settings remains to be determined. However, in a mouse models of multiple sclerosis, A-MSCs were found to be more potent in inhibiting disease due to their broader expression of homing molecules (Payne et al., 2013). Thus, aside from proprietary concerns motivating the commercial use of A-MSCs, comparative analysis of A-MSCs and BM-MSCs from the same donors has indicated that A-MSCs may have increased immunomodulatory capacity (Menard et al., 2013).

MSC isolation from the bone marrow or adipose tissue is, however, associated with contamination from cell types inhabiting the anatomical region of the source tissue (Xu et al., 2010; Schneider et al., 2017). Of the cells that compose the adipose stromal-vascular cell fraction, stromal fibroblasts, and dermal fibroblasts are plastic adherent and may persist alongside cultured A-MSCs (Phinney et al., 1999; Blasi et al., 2011). Furthermore, the growth kinetics, differentiation potential, and immunogenicity of isolated BM-MSCs and A-MSCs can vary depending on donor age and health (Siegel et al., 2013; Choudhery et al., 2014). A-MSCs isolated from aged rats failed to elicit T cell suppression while BM-MSC mediated immunosuppression was noted to be more effective in young rats (Wu et al., 2014). A-MSCs derived from obese and type 2 diabetes patients were also less effective in suppressing lymphocyte proliferation and activating M2 macrophage phenotype (Serena et al., 2016). Therefore, although the ease of accessibility, greater yield, and immunosuppressive qualities of A-MSCs make them more suited to clinical application, caveats relating to MSC purity and donor health must be considered.

## Immunomodulatory Properties of MSCs

Part of the initial excitement with using MSCs as a therapeutic product resulted from their supposedly immune privilege status as MSCs do not express major histocompatibility complex (MHC) molecules involved in immune recognition (Le Blanc et al., 2003). This meant that MSCs could be expanded as an off-the-shelf, allogeneic product, and be administered to patients across MHC barriers (i.e., transplantable between HLA-mismatched patients), which is commercially attractive and clinically practical. However, it became apparent that MSCs do express MHC class I constitutively and upregulate MHC class II in the presence of inflammatory cues (Tse et al., 2003). Moreover, repeated injections of MSCs can elicit antibodies and lead to sensitization and rejection (Eliopoulos et al., 2005; Badillo et al., 2007; Campeau et al., 2009; Zangi et al., 2009). MSCs are susceptible to lysis by allogeneic CD8^+^ T cells and NK cells (Crop et al., 2011). Recent findings have also indicated that injected MSCs are killed by cytotoxic T and NK cells in a tissue environment rich in these cells (Galleu et al., 2017). Nevertheless, despite the lack of cell differentiation or sustained engraftment in injured tissues, it was clear that MSC treatment led to resolution of inflammation.

### Effects on Adaptive Immunity

In the early 2000s, studies demonstrated that BM-MSCs dampen T cell proliferation *in vitro* and *in vivo*, in response to polyclonal stimuli (Bartholomew et al., 2002; Di Nicola et al., 2002). This was soon followed by the demonstration that MSCs can inhibit T cell proliferation, interferon-gamma (IFN-γ) production, and cytotoxic activity in response to antigen-specific stimuli, but do not require MHC molecules or antigen presentation by antigen presenting cells (Krampera et al., 2003). When co-cultured with alloreactive T cells, MSCs can directly induce the proliferation of Foxp3^+^ regulatory T (Treg) cells, specialized T cells with immunosuppressive activity that help maintain tolerance to tissue antigens (Selmani et al., 2008). MSCs have also been shown to generate Treg cells by inducing the expression of Foxp3 in T cells and inhibiting their differentiation to Th17 cells, another T cell subset with inflammatory activity (Ghannam et al., 2010).

As B cell responses are mainly dependent on T cell help, inhibition of T cell function by MSCs can impair B cell function and humoral immunity. In murine co-culture experiments of MSCs with purified B cells, MSCs were shown to also directly inhibit B cell proliferation and differentiation into antibody-producing effector B cells (Augello et al., 2005; Asari et al., 2009). Co-cultures of MSCs with human B cells, on the other hand, have yielded conflicting results, with some studies showing inhibitory effects on antibody production and chemotactic properties (Corcione et al., 2006), while others showed that MSCs can promote B cell function by supporting B cell survival, expansion and differentiation (Traggiai et al., 2008), and antibody secretion (Rasmusson et al., 2007).

The initiation of adaptive immune responses depends crucially on dendritic cells (DCs), which survey the skin and mucosal tissues, capturing and processing antigens for display to T cells in an MHC-restricted manner. MSCs have been shown to interfere in the differentiation of monocytes to DCs (Nauta et al., 2006; Spaggiari et al., 2009), and inhibit the upregulation of MHC class II and co-stimulatory molecules associated with DC maturation and antigen presentation (Zhang et al., 2004) to skew their phenotype to an immature state (Zhang et al., 2009). MSCs have also been shown to reduce the capacity of DCs to activate alloreactive T cells (Zhang et al., 2004), modulate their cytokine secretion profile towards production of anti-inflammatory molecules, such as interleukin (IL)-10, and block the release of pro-inflammatory cytokines, such as tumor necrosis factor-alpha (TNF-α), IFN-γ, and IL-12 (Aggarwal and Pittenger, 2005).

### Effects on Innate Immunity

MSCs also interact with the innate immune system by conferring immunomodulatory effects on other immune cell types, including monocytes, macrophages, neutrophils, and natural killer (NK) cells (Le Blanc and Mougiakakos, 2012).

Monocytes and macrophages form the mononuclear phagocyte system and are essential components of inflammation and tissue repair (Jung, 2018). Blood monocytes that enter inflamed sites in the body respond to local inflammatory stimuli and differentiate into monocyte-derived cells that resemble macrophages or DCs (Teh et al., 2019). At early stages of inflammation, tissue-infiltrating monocytes secrete pro-inflammatory TNF-α and IL-1, while monocytes found at later stages of inflammation exhibit anti-inflammatory properties (Teh et al., 2019). Macrophages exhibit similar plasticity in their phenotype and function in response to signals in the local microenvironment, differentiating either into M1 macrophages that release pro-inflammatory factors (e.g., IFN-γ and TNF-α) or M2 macrophages that promote tissue repair by secreting anti-inflammatory factors (e.g., IL-10 and transforming growth factor (TGF)-β) (Biswas and Mantovani, 2010; Murray and Wynn, 2011). While recognized as an overly simplified classification scheme, polarization of monocytes and macrophages is evident in studies reporting MSC-mediated resolution of tissue injury. In particular, MSCs produce IDO and prostaglandin E_2_ (PGE_2_), which polarise macrophages toward an M2 phenotype that is characterized by secretion of IL-10 (Németh et al., 2009; François et al., 2012; Melief et al., 2013a). MSC-driven polarization of macrophages has been reported to underlie the immunomodulatory effects of MSC therapy in various disease models, including sepsis (Németh et al., 2009), wound healing (Zhang et al., 2010) and renal ischemia-reperfusion injury (Li et al., 2013).

The interactions between MSCs and monocytes/macrophages are bidirectional, as several studies have shown that MSCs are activated by inflammatory cytokines produced by macrophages at early stages of inflammation. For example, in a murine model of sepsis, MSC treatment attenuated disease by inducing IL-10 production by macrophages (Németh et al., 2009). This increase in IL-10 production was dependent on PGE_2_ secretion by MSCs, which was in turn dependent on TNF-α and iNOS signalling from the macrophages. Similarly, in a mouse model of zymosan-induced peritonitis, inflammatory cytokines secreted by peritoneal macrophages activated human MSCs to produce TNF-α–stimulated gene 6 protein (TSG-6), which in turn inhibited NF-κB signaling in macrophages and attenuated the release of inflammatory cytokines in a negative feedback loop (Choi et al., 2011). The central role of macrophages in MSC therapy has been demonstrated in several disease models, including sepsis (Németh et al., 2009), allergic asthma (Mathias et al., 2013) and GvHD (Galleu et al., 2017), whereby the beneficial effects of MSCs were abrogated in the absence of macrophages.

Recent studies have linked the immunosuppressive effects of MSC treatment to the phagocytic properties of monocytes and macrophages. Lung entrapment of intravenously administered MSCs is a well-documented phenomenon (Fischer et al., 2008; Kidd et al., 2009; Lee et al., 2009; Eggenhofer et al., 2012; Mathias et al., 2013). Entrapped MSCs are phagocytosed by circulating monocytes, neutrophils, and lung macrophages, which adopt an immunoregulatory phenotype and may elicit non-specific immunosuppressive effects (Galleu et al., 2017; de Witte et al., 2018).

Neutrophils, being the most abundant innate immune cells, are the first responders to microbial challenge and accumulate at the wound site within minutes of injury (Joel et al., 2019). MSCs have been shown to enhance neutrophil phagocytic activity, aiding pathogen clearance (Hall et al., 2013). Since neutrophils are non-proliferative cells with a short lifespan, their survival is pivotal to their role in pathogen elimination (Luo and Loison, 2008). Through constitutive release of IL-6, MSCs act to inhibit apoptosis of neutrophils (Le Blanc and Mougiakakos, 2012), extending their lifespan and providing an enhanced opportunity for pathogen elimination and tissue repair to take place. MSCs express functional Toll-like receptors (TLRs), which recognize “danger” signals and activate immune responses to fight infection or resolve inflammation (Hwa Cho et al., 2006; Pevsner-Fischer et al., 2007; Tomchuck et al., 2008). Activation of TLR3 on MSCs enhanced neutrophil viability and function (Cassatella et al., 2011). Similarly, TLR-activated BMMSCs promoted the survival of resting and activated neutrophils through the production of IL-6, IFN-β, and GM-CSF (Hirano et al., 2000; Raffaghello et al., 2008). Although neutrophils have the capacity to phagocytose apoptotic MSCs, how this relates to the immunomodulatory effects of MSC therapy remains to be clarified, particularly in view of the short lifespan of neutrophils.

NK cells mediate innate immunity by recognizing and lysing cells that are unable to display or have downregulated MHC class I molecules, such as tumor cells (Malmberg et al., 2017). When co-cultured with MSCs, IL-2-activated NK cells downregulated their expression of activating receptors, NKp30 and NKp44, and NKG2D, produced less IFN-γ, and exhibited decreased cytotoxicity to tumor cells (Spaggiari et al., 2008).

**Figure 1:**
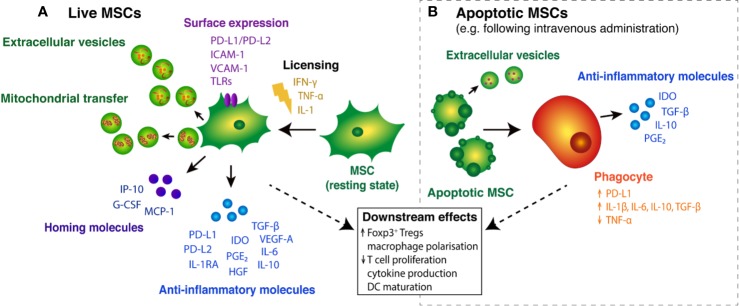
Immunosuppressive effects of live and apoptotic MSCs. **(A)** In the presence of pro-inflammatory cytokines, resting MSCs become “licensed” to secrete key anti-inflammatory molecules, including PD-L1/PD-L2 (Davies et al., 2017), TGF-β (Niu et al., 2017) and IDO (Kim et al., 2018). Licensed MSCs also secrete homing molecules that promote MSC migration and Treg cell recruitment to tissue injury sites (Yu et al., 2011; Lunardi et al., 2014). Surface expression of various molecules on MSCs mediates interactions with T cells and guides MSC migration into inflammatory tissues (Ren et al., 2008; Chinnadurai et al., 2014). MSC cargo, in the form of extracellular vesicles (EVs) and subcellular components, such as mitochondria, may also play a role in MSC-mediated immunosuppression. **(B)** Following intravenous administration, MSCs can become apoptotic and are engulfed by circulating phagocytes, triggering the expression and release of immunomodulatory molecules (Galleu et al., 2017; de Witte et al., 2018; Cheung et al., 2019). Apoptotic cells can secrete immunosuppressive cargo packaged in extracellular vesicles (Caruso and Poon, 2018). Together, the host response elicited by live and engulfed MSCs leads to broader downstream effects on immune cell function.

The plethora of studies demonstrating that MSCs exert potent immunomodulatory capacity prompted a shift in the focus of the field, away from utilizing their differentiation potential to harnessing their capacity to modulate immune function. This immunomodulation of various effector functions seems necessary for allogeneic MSCs to establish a tolerogenic environment that can grant MSC-specific anti-inflammatory and reparative processes to take place. The precise mechanistic pathways that lead to this tolerogenic environment are yet to be delineated; however, it is apparent that MSCs modulate the immune system *via* direct cell contact and an indirect mechanism through the production and secretion of soluble factors (Uccelli et al., 2008) (**Figure 1**).

### Cell Contact-Dependent Immunomodulation

The involvement of cell-to-cell contact in MSC immunomodulation was made evident in transwell experiments in which MSCs and T cells were physically separated by a membrane. MSCs inhibited allogeneic T cell proliferation in transwells, which was further reduced when MSCs and T cells were co-cultured together (Di Nicola et al., 2002). This indicated that the immunosuppressive effects of MSCs in a mixed lymphocyte reaction are due predominantly to soluble factors and are greatly enhanced by contact with their target cells. Cell contact is facilitated by various chemokines and adhesion molecules expressed by MSCs upon activation by inflammatory cytokines (Castro et al., 2019). For example, MSCs express high levels of CXCL9, CXCL10, and CXCL11 in response to inflammatory cytokines (Ren et al., 2008). These potent T cell attractants bind to CXCR3 on activated T cells, and antibody blockade of CXCR3 binding inhibited T cell chemotaxis toward MSCs and abrogated the inhibitory effects of MSCs (Ren et al., 2008). In another study, activated T cells induced the expression of adhesion molecules, ICAM-1 and VCAM-1, on MSCs, which was positively correlated with the immunosuppression of various T lymphocyte subsets (Ren et al., 2010). Accordingly, genetic deletion of both adhesion molecules in MSCs led to a significant decrease in their immunosuppressive capacity (Ren et al., 2010).

The inhibitory effects of mouse MSCs on antigen-specific T cell activation were also greatly reduced in transwell experiments (Krampera et al., 2003). The requirement for cell contact suggests that MSCs act to directly inhibit T cell activation. Indeed, contact-dependent inhibition of T cell activation was demonstrated to occur *via* ligands expressed by human and mouse MSCs that bind to programmed cell death protein-1 (PD-1) on activated T cells to provide an inhibitory signal (Augello et al., 2005; Chinnadurai et al., 2014). However, it should be noted that PD-1/PD-L1 inhibition of T cell activation by MSCs can occur independent of cell contact, as human MSCs also secrete PD-1 ligands (PD-L1 and PD-L2) constitutively and in response to inflammatory cytokines (Davies et al., 2017).

### Immunomodulation by Soluble Factor Secretion

MSCs separated from effector cells in transwell experiments exhibited reduced, rather than total loss of, immunosuppressive effects on T lymphocyte proliferation, indicating that MSCs exert effects through the secretion of soluble factors, such as cytokines, growth factors, and chemokines, in addition to direct cell contact (Di Nicola et al., 2002). In the past 15 years, a plethora of studies have investigated the effects of MSCs on cell-mediated and humoral responses in the innate and adaptive immune system. These studies have identified a broad range of soluble factors that are critical for MSC-mediated immunosuppression. The array of mechanisms employed by MSCs may reflect the heterogeneous composition of cells in current MSC preparations. The current view is that, while MSCs employ both cell-cell contact and soluble factors for robust pleiotropic immunomodulation, primary immunosuppressive effects are exerted *via* cytokines *in vivo*. Importantly, in inflammatory conditions, MSCs have been shown to utilize signals from the immediate cytokine milieu to fine-tune their immunosuppressive effects for tissue repair and wound healing, according to the required intensity, duration, and site of inflammation resolution (Kusuma et al., 2017).

#### Pro-Inflammatory Cytokines—For Priming MSC Immunosuppression

It is well accepted that immunosuppression is not an inherent feature of MSCs but rather a result of activation, or “priming,” by an inflammatory environment (Krampera et al., 2006; English et al., 2007; Hemeda et al., 2010; Ren et al., 2010). Upon T cell activation, IFN-γ is released and continues to promote T cell activation and expansion. However, in the presence of MSCs, IFN-γ binds to its receptor on MSCs and results in in the suppression of T cell proliferation (Krampera et al., 2006). This effect has been confirmed by IFN-γ receptor-negative MSCs that fail to inhibit T cell proliferation (Ren et al., 2008). In addition, IFN-γ levels serve to regulate MSC proliferation and differentiation *via* IDO secretion (Croitoru-Lamoury et al., 2011). Similarly, TNF-α “primes” MSCs, which in turn upregulates a host of immunosuppressive factors that may, for example, contribute to tissue repair mechanisms (Ren et al., 2010).

#### Anti-Inflammatory Cytokines—For Driving MSC Immunosuppression

MSCs secrete an array of cytokines that have immunoregulatory effects. A key regulatory factor secreted by IFN-γ-primed MSCs is IDO (Kim et al., 2018). IDO is a rate-limiting enzyme of tryptophan catabolism, resulting in decreased levels of this enzyme (Grohmann et al., 2003). Since tryptophan is required for T cell proliferation, its depletion leads to T cell suppression (Yang et al., 2009) *via* direct (Meisel et al., 2004) and indirect pathways (François et al., 2012). In addition, IDO induces Treg cells *in vitro* and is responsible for B cell growth arrest and apoptosis (Maby-El Hajjami et al., 2009). With increasing Treg cell levels during MSC-mediated immunosuppression (Erkers et al., 2013; Hsu et al., 2013), there is a stimulation of IL-10 production (Engela et al., 2013), a cytokine that has been associated with inflammation resolution.

In order to confer their anti-inflammatory effects, MSCs may need to home to the site of injury (Kean et al., 2013). This homing is made possible by a range of soluble factors operating to ensure MSCs reach the appropriate site of tissue injury (Musiał-Wysocka et al., 2019). Vascular endothelial growth factor (VEGF)-A is known to stimulate angiogenesis *via* promotion of endothelial cell survival, proliferation, migration, and differentiation (Shibuya, 2011; Ge et al., 2018). IL-8-induced VEGF production by MSCs leads to increased angiogenesis and allows MSCs to utilize these blood vessels to reach the injury site (Hou et al., 2014). Interferon gamma induced protein (IP)-10 secretion by MSCs recruits Treg cells to sites of inflammation, resulting in an immunosuppressive microenvironment (Lunardi et al., 2014). IP-10 production also induces MSC migration to inflammatory sites (Rice and Scolding, 2010). Additionally, paracrine release of monocyte chemoattractant protein (MCP)-1 by MSCs enables MSC migration towards tissue injury sites (Boomsma and Geenen, 2012) and induces Fas ligand-dependent apoptosis of lymphocytes (Akiyama et al., 2012). Granulocyte-colony stimulating factor (G-CSF) release by MSCs increases both their mobility into peripheral blood systems and homing to the site of injury (Yu et al., 2011). Intracellular adhesion molecule-1 (ICAM-1) and vascular adhesion molecule-1 (VCAM-1) are both vital for the activation, rolling, and transmigration of leukocytes in immune responses (Musiał-Wysocka et al., 2019). Upregulation of ICAM-1 and VCAM-1 on the surface of MSCs has been shown to mediate MSC homing to the secondary lymphoid organs, allowing MSC-T cell interactions to take place (Ren et al., 2010). These interactions, in turn, lead to suppression of T cell proliferation (Ren et al., 2010).

Another notable regulatory factor secreted by MSCs is PD-L1. Secretion of PD-L1 by MSCs suppresses CD4+ T cell activation, downregulates pro-inflammatory IL-2 secretion, and suppresses T cell proliferation and cytokine production (Davies et al., 2017). PD-L1 also regulates Treg cell function, thus inhibiting pro-inflammatory T cell responses (Francisco et al., 2009). MSCs also produce PGE_2_, a lipid mediator that acts *via* paracrine mechanisms to alter several arms of the immune system (Castro et al., 2019). PGE_2_ release suppresses T cell activation and proliferation, both *in vitro* and *in vivo* (Aggarwal and Pittenger, 2005; Najar et al., 2010). It has also been shown to bind to CD4^+^ T cells in order to inhibit Th17 differentiation (Duffy et al., 2011). In addition, MSC-secreted PGE_2_ inhibits DC maturation (Spaggiari et al., 2009) and induces a shift in M1 macrophages to adopt a M2 phenotype (Vasandan et al., 2016). IL-6 release by MSCs inhibits MSC differentiation and protects it from apoptosis in a paracrine manner (Pricola et al., 2009). IL-6 also enhances plasma interleukin-1 receptor antagonist (IL-1RA) and IL-10 release by MSCs *in vivo* (Steensberg et al., 2003). Another important soluble factor secreted by MSCs is TGF-β which acts to inhibit T cell proliferation, differentiation, and effector functions in a soluble manner and *via* direct cell contact (Kong et al., 2009; Niu et al., 2017). Furthermore, it promotes the conversion of naïve CD4^+^ T cells to Treg cells (English et al., 2009). Other MSC-secreted cytokines like hepatocyte growth factor (HGF) mediate anti-inflammatory, anti-apoptotic, and antifibrotic mechanisms to resolve inflammation (Kennelly et al., 2016). It is apparent from accumulative studies that there are several cytokines operating in redundancy to ensure that MSC-mediated immunosuppression is established in times of tissue injury, infection, and trauma.

### MSC Licensing

Importantly, to become immunosuppressive, MSCs need to be activated, or primed, by inflammatory cytokines in a multistep process called licensing (Krampera, 2011). MSC activation is mediated primarily by IFN-γ, which is one of the first cytokines produced upon T cell activation (Polchert et al., 2008; Ren et al., 2008). Blocking IFN-γ receptor with neutralizing antibodies was shown to abolish the immunomodulatory capabilities of human MSCs (Krampera et al., 2006). Similarly, MSCs isolated from knockout mice that were unable to respond to IFN-γ were incapable of inhibiting lymphocyte proliferation (Ren et al., 2008). Although the presence of IFN-γ is enough to prime MSCs, the combination of IFN-γ and either TNF-α, IL-1α, or IL-1β greatly enhances the inhibitory effects of MSCs (Ren et al., 2008).

The requirement for MSCs to be activated by inflammatory signals may explain why MSCs were only effective in treating graft-versus-host disease (GvHD) after inflammation had been established but did not show immunomodulatory properties when infused before inflammation was present (Sudres et al., 2006). In this context, differential triggering of TLRs on MSCs induces modulation of their immunosuppressive potency, with TLR-3 activation promoting an anti-inflammatory phenotype, whereas activation by TLR-4 promotes a pro-inflammatory phenotype (Waterman et al., 2010). Thus, MSCs can act either as a suppressive or pro-inflammatory cell, and this immune plasticity or functional polarization can be driven by the ligand, kinetics, and strength of the TLR stimulation (Krampera, 2011).

## Live Versus Apoptotic MSCs

The efficacy of MSCs in various preclinical models of inflammatory diseases is well documented. In these settings, MSCs are exposed to pro-inflammatory cytokines, which are reported to “license” MSCs (e.g., IFN-γ, TNF-α, and TLR activation), but can also induce cell death (Salaun et al., 2007; Li et al., 2019). MSCs are also susceptible to activated NK cell-mediated killing *via* tumor necrosis factor-related apoptosis-inducing ligand (TRAIL) and Fas ligand (FasL) pathways (Spaggiari et al., 2006; Götherström et al., 2011).

A series of recent studies has indicated that MSC survival in the inflamed tissue may not be pertinent for the manifestation of MSC-mediated immunosuppression. In fact, apoptotic MSCs can confer immunosuppressive effects upon their administration into inflammatory sites *in vivo* (Galleu et al., 2017), suggesting that cell viability does not necessarily correlate with therapeutic efficacy. Recent studies have linked MSC apoptosis with their therapeutic effects in animal models of GvHD, sepsis, acute lung injury, and allergic airway inflammation (Luk et al., 2016; Galleu et al., 2017; Laing et al., 2018). The clinical response to MSC therapy in GvHD patients directly correlates with the ability of their immune cells to induce MSC apoptosis (Galleu et al., 2017). Whether the immunomodulatory effects in MSC-based therapies are directly mediated by factors produced by apoptotic MSCs or *via* the host response to apoptotic MSCs remains to be established. Furthermore, most MSCs are cleared shortly after infusion, with limited evidence of engraftment. The rapid clearance of these cells has been attributed to apoptosis (Eggenhofer et al., 2012) and this may be orchestrating local immune responses that lead to the anti-inflammatory effects seen as part of MSC administration (de Witte et al., 2018; Cheung et al., 2019). Although these findings challenge the longstanding view that viable MSCs are critical for therapeutic efficacy, studies have also shown limited efficacy with fixed or necrotic cells (Gupta et al., 2007; Németh et al., 2009; Kavanagh and Mahon, 2011; Mathias et al., 2013), suggesting that MSCs are most efficacious when viable at the time of administration.

### MSC-Derived Extracellular Vesicles

Recent efforts in dissecting the mechanisms of MSC therapy have focused on the role of extracellular vesicles (EVs) as biological modulators. Cells produce three main types of EVs — exosomes (50–100 nm in diameter) and microvesicles (0.1–1 µm in diameter) produced by healthy cells, and apoptotic bodies produced by apoptotic cells (Caruso and Poon, 2018). Exosomes have the capacity to influence several aspects of immunity by activating or suppressing cytokine secretion, immune cell differentiation and polarization and T cell activation (Phinney et al., 2015; Chen et al., 2016). Exosomes derived from healthy MSCs in culture have been found to have anti-inflammatory effects in human disease models (Del Fattore et al., 2015; Anderson et al., 2016; Chen et al., 2016). Apoptotic cells also produce exosomes which have important immunomodulatory function such that they form a means through which dying cells communicate with their surroundings to bring about the anti-inflammatory effects (Caruso and Poon, 2018). To establish a therapeutic platform based on the delivery of MSC-derived exosomes would require a greater understanding of the quantity and quality of exosomes derived from both viable and apoptotic cells. Additionally, a greater understanding of exosomes in various disease settings is required since each disease varies in its profile, key players, and the nature of manifestation. Despite these gaps, it is evident that exosome-based MSC therapy would be an alternative drug delivery system that would circumvent the costs and complexities associated with propagation of whole cells.

### Mitochondria in Secreted EVs

Since mitochondria regulate the energy metabolism of a cell, the health and state of mitochondria will have a direct impact on oxidative stress and cell death (Guo et al., 2013). Therefore, it becomes evident that mitochondria can impact MSC immunosuppression. Mitochondrial transfer has been shown to pivotal in the therapeutic efficacy of MSCs in various pre-clinical models, such as brain injury, cardiac myopathies, acute ARDS, and chronic respiratory disorders (Li et al., 2014; Jackson et al., 2016; Torralba et al., 2016; Morrison et al., 2017). Mitochondria can be released as part of EVs in a functionally active state that enhances oxidative phosphorylation and dampens oxidative stress in recipient cells (Torralba et al., 2016). Overall, this leads to repair and healing of injured and inflamed sites. As part of MSC therapy, it is vital to reduce mitochondrial dysfunction that causes pathophysiology and strive to utilize healthy mitochondria to drive anti-inflammatory functions. Despite preliminary evidence and understanding of the significant role that mitochondria plays at the cellular level, the precise mechanisms by which mitochondria eject as part of EVs remains to be uncovered. In addition, an understanding of how EV-packaged mitochondria is taken up by recipient cells will be key in tailoring MSC therapy around the bioenergetics of this organelle.

## Therapeutic Applications of MSCs

There is much clinical interest in utilizing the immunomodulatory properties of MSCs in cellular therapy. Several MSC products have already been approved for various clinical applications with many others undergoing investigation in clinical trials. *Cartistem* is licensed for treatment of degenerative arthritis in South Korea, *Cupistem* and *Alofisel* for treatment of Crohn's anal fistula in South Korea and Europe, respectively, TEMCELL as an acute GvHD treatment in Japan, and *Prochymal* for the same indication in Canada and New Zealand (Gao et al., 2016; Galipeau and Sensebe, 2018).

Clinical use of MSCs necessitates large-scale expansion that cannot be sustained through tissue culture dishes or flasks in a laboratory setting. A constant supply of high cell numbers requires robust and economically viable culture processes. Meanwhile, risks that may compromise clinical use — such as cell transformation, secretion aberrations, and xenogeneic contact (e.g., animal serum) — must be reduced. To improve the feasibility of clinical use, there must be compromise between obtaining high cell numbers while ensuring the MSC immunophenotype is unaltered.

Another significant aspect of MSC therapy revolves around utilization of “frozen” or cryopreserved versus fresh MSCs. It is common practice for fresh MSCs to be used in preclinical models versus the predominant use of cryopreserved cells in the clinical setting (Moll et al., 2016). This contrasting practice has led to discrepancies in the protective effects of MSCs as outlined in the literature, compared to clinical outcomes observed in patients with MSC therapy. To date, it has been well documented that MSC potency can be affected by tissue origin, culture conditions, and modes of cell delivery, including the use of fresh versus thawed cells (Galipeau, 2013; Marquez-Curtis et al., 2015). Furthermore, upon recovery from cryostorage, thawed cells show various changes in molecular and physical integrity compared to fresh cells that may also impact immunomodulatory properties of MSCs when used from cryopreservation rather than fresh (Moll et al., 2014; Chinnadurai et al., 2016). The choice between the two will impact how MSC therapy products should be developed and whether an “off-the-shelf” approach would allow for therapeutic effects to be delivered without compromising the potency and immunomodulatory profile of the cell product.

## MSC Culturing Systems

### Cell Culture Supplements

**Figure 2:**
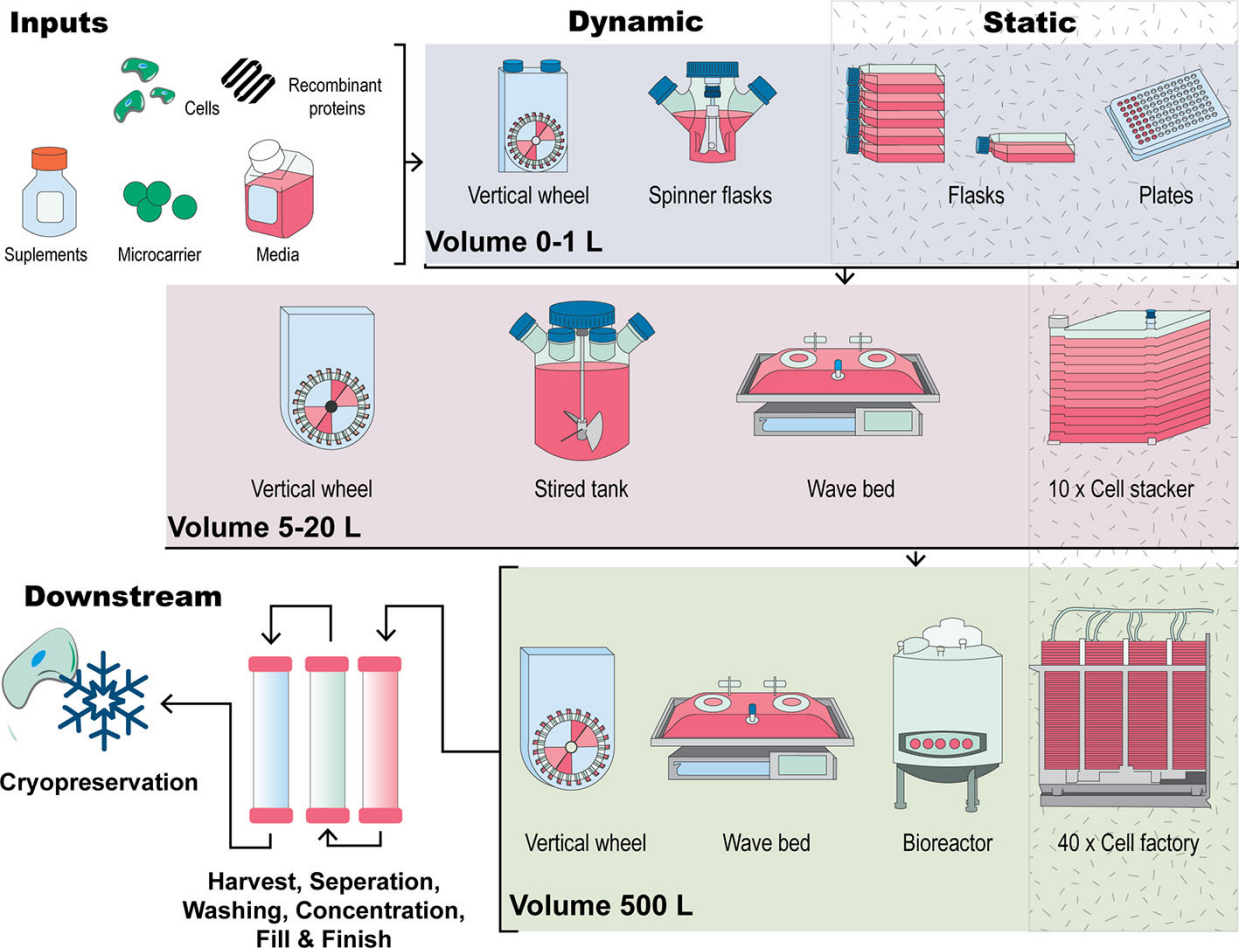
A visual comparison of expansion strategies for human MSCs. Inputs to the process include cells, media, supplements, a culture surface (flask, cell stacker, microcarriers) and other additives including growth factors. Figure adapted from (Kropp et al., 2017). Traditional culturing methods encompass 2D, planar technologies such as expanding MSCs in a culture dish or flask by continual passaging. Scale out of this approach uses cell stackers or multilayered flasks which work in this manner through multiplication of the culturing flask. In comparison, scale-up manufacturing methods involve MSCs forming aggregates or being seeded onto microcarrier in suspension in bioreactor systems such as stirred tank, vertical wheel or wave bag bioreactors. Downstream processes such as cell harvesting cell washing, cell concentration, finish and fill and storage through cryopreservation are also critical parts of the manufacture of MSCs for clinical applications.

A regulatory requirement for the therapeutic use of cells is that they are manufactured under a quality system or using Good Manufacturing Practice (GMP) (Abbasalizadeh et al., 2017). In this system, all inputs to the process (media, supplements, growth factors) also need to be manufactured under GMP conditions, which include reagent validation, batch testing, and release under appropriate release criteria before use. This means that when considering all of the parts of a manufacturing process, an ability for the bioreactor to be utilized under a quality system is imperative if the cells produced are to be used clinically. A number of cell culture systems meet these criteria and are used currently (see **Figure 2**). Inputs to these culture systems include cells, media, supplements (often animal-derived serum) and growth factors. Synthetic media (serum-free or xeno-free) media typically have the molecules to support cell growth already included in the media but may require pre-coating of the growth surface with recombinant proteins or fragments which support cell attachment.

Serum (usually bovine or human) is included in MSC expansion media to provide nutrients for growth, attachment-promoting proteins (e.g., fibronectin, and vitronectin) for cell adhesion, and hormones and lipids to stimulate cell proliferation *in vitro* (Oikonomopoulos et al., 2015). However, the use of fetal bovine serum (FBS) has recently raised concerns that animal proteins and peptides may contaminate human MSCs during culture (Gregory et al., 2006). This could lead to viral or prion transmission and cause aberrant immune reactions in a clinical setting. In some cases, antibodies to FBS proteins have been detected in clinical settings where transplanted cells have been exposed to FBS (Horwitz et al., 2002; Sundin et al., 2007). In addition, there are ethical concerns associated with the use of FBS (Tekkatte et al., 2011). Further issues involve batch to batch variability and the requirement for extensive qualification of FBS for cell manufacturing purposes (Witzeneder et al., 2013). The limited supply and high cost of FBS is also a limitation in the application of cell therapies (Fang et al., 2017). For example, estimates of FBS availability indicate that in the future it is unlikely that supply will keep up with demand, particularly given that FBS is a by-product of the meat industry (Karnieli et al., 2017). Cell culture supplementation with human serum (both allogeneic and autologous) has been studied (Gottipamula et al., 2013) and the use of pooled human AB serum (hABS) is becoming increasingly widespread, at least in *in vitro* studies. In one such study, use of hABS was found to significantly enhance MSC expansion in 2D cultures compared to FBS and had similar immunosuppressive effects (Thaweesapphithak et al., 2019). In this study, hABS was also used in the isolation of MSCs from tissue and cryopreservation. Savelli et al. (2018) cultured MSCs in a hollow fiber, perfused bioreactor and found that a particular population of cells, the mesodermal progenitor cells (MPCs), were enriched compared to cultures in media supplemented with FBS, where only a MSC phenotype was observed. Supplementation with human AB serum was tested in a comparative study of MSC expansion in planar and microcarrier culture at reasonable scale (2 L stirred tank systems utilizing microcarriers) (Tozetti et al., 2017). The microcarrier-based systems were found to give significantly greater cells/cm^2^ than planar systems, however efficient harvesting was identified as a hurdle to obtaining maximum cell yields. Of course, there are some limitations such as the amount that can be supplied and the risk of spreading previously unknown or new human pathogens (Karnieli et al., 2017).

A common alternative for large-scale MSC manufacture is human platelet lysate (HPL) prepared under a quality system or good manufacturing practice (GMP) guidelines. HPL provides strong growth-promoting activity to support the expansion of a variety of cells (Choi et al., 1980; Eastment and Sirbasku, 1980; Hara et al., 1980). In fact, there are now ample studies demonstrating that proliferation of MSCs from various tissue sources is higher when HPL is used (Schallmoser et al., 2007; Bieback et al., 2009; Gottipamula et al., 2012; Gottipamula et al., 2013; Gottipamula et al., 2016; Czapla et al., 2019; Kakudo et al., 2019) and generally studies utilizing HPL for *in vitro* expansion of MSCs have found it to be an acceptable alternative to FBS in terms of maintaining cellular features for clinical applications (Fekete et al., 2012; Becherucci et al., 2018). However, studies on the effects of HPL on the immunosuppressive capacity of MSCs have been contradictory. In one study, HPL-expanded MSCs displayed altered expression of surface molecules, impaired lymphocyte, and natural killer cell suppression when compared to FBS (Abdelrazik et al., 2011). In another study, a higher immunosuppressive effect was observed for BM-MSCs expanded in HPL-supplemented media (Gottipamula et al., 2012). Other studies comparing cell expansion in HPL- or FBS-supplemented media have reported no difference in the immunosuppressive effects of BM-MSCs (Bieback et al., 2009), or in the secretion profiles of A-MSCs (Czapla et al., 2019). Chromosomal stability appeared to be the same if not better for cells grown in HPL (Shih and Burnouf, 2015; Astori et al., 2016). Although considered a safe tool for clinical expansion purposes, there are limitations to the use of HPL as an FBS alternative for MSC expansion. Given the current literature is unclear on the consensus effects of HPL on MSC immunosuppression, further research is required to clarify the effects (if any) of HPL on the immunosuppressive capacity of MSCs *in vivo*. There have been a number of clinical studies involving MSCs that have been expanded using HPL as the supplement for MSC production, the result of which have indicated that HPL can safely replace FBS for clinical-scale MSC manufacture (von Bonin et al., 2009; Centeno et al., 2011; Introna et al., 2014; Bieback et al., 2019). In addition, a recent survey of European centers manufacturing cells for GvHD survey of showed that 77% of the centers were using HPL in preference to FBS (which was mostly supplemented at 5% in media) (Trento et al., 2018).

There is an increasing number of synthetic cell culture media available commercially, optimized for MSCs to avoid the issue of batch-to-batch variability of biologically derived media supplements. These media typically do not contain animal- or human-derived supplements and can be described as serum- or xeno-free (SF or XF). For example, Gottipamula et al. compared the growth kinetics, cell surface markers, morphology, differentiation potential, and immunosuppressive properties of BM-MSCs expanded in small volume cultures in a range of SF and XF media and one media was also used in a 10-layer cellSTACK® (Gottipamula et al., 2016). Cell yields were lower in the cellSTACK®, compared to FBS media highlighting that scaling up production even from small to moderate scale can present some challenges. Optimization may need to be carried out at each scale tested. These media are still rather expensive (approx. the same as FBS and HPL per unit volume) meaning that they are not currently being used to expand cells for clinical application to our knowledge. Costs are expected to reduce as with the economies of scale associated with more widespread use. A summary of the relative advantages and disadvantages of each media supplement type discussed above is presented in **Table 1**, particularly for human-derived and synthetic media supplements over animal-derived supplements such as FBS.

**Table 1:** Summary of cell culture media growth supplements commonly used; fetal bovine serum (FBS), pooled human AB serum (hABS), human platelet lysate (HPL), and synthetic media and their relative advantages and disadvantages in a cell therapy context.

Supplement	Advantages	Disadvantages
Fetal Bovine Serum (FBS)	Long history of use	Limited supply (Fang et al., 2017)
	Extensive clinical experience	Animal disease transmission to humans (Gregory et al., 2006)
		Possible immune response (Horwitz et al., 2002)
		Less preferred from regulatory viewpoint (Karnieli et al., 2017)
		Batch-to-batch variability, requiring qualification (Mendicino et al., 2014)
		High cost
		Ethical concerns (Tekkatte et al., 2011)
Pooled hAB Serum (hABS)	Human origin	Limited supply
	Universal donor - meets most HLA requirements	Relies on donation
	Appears to have a higher proliferative capacity (Thaweesapphithak et al., 2019)	Ethical issues associated with use of human-derived products (Jacobs et al., 2019)
	GMP grade available	Potential spread of human diseases (Karnieli et al., 2017)
Human platelet lysate (HPL)	Human origin	Limited supply
	Higher proliferative capacity established (Bieback et al., 2009; Kakudo et al., 2019)	Relies on donation
	GMP grade available	Ethical issues associated with use of human-derived products (Jacobs et al., 2019)
	Widely used clinically (77% centers in Europe) (Trento et al., 2018)	Potential spread of human diseases
	Chromosomal stability (Juhl et al., 2016)	
	Enhanced MSC immunosuppressive effects observed (Gottipamula et al., 2012)	Still some debate on effect on MSC immunomodulatory effects (Abdelrazik et al., 2011)
Synthetic media	Potentially unlimited supply	Use with cells for clinical studies not established (Lensch et al., 2018)
	Chemically defined	Expensive
	Higher proliferative capacity (Patrikoski et al., 2013)	Proliferative capacity dependent on cell type/origin, optimized media composition (Cimino et al., 2017)
	Minimal batch-to-batch variability (Cimino et al., 2017)	May rely on animal-derived or recombinant cell adhesion molecules

### 2D Versus 3D Culture of MSCs for Therapeutic Applications

Traditionally, undifferentiated MSCs are maintained and expanded at low density in two-dimensional (2D) monolayer conditions in culture vessels with planar surfaces, with cells adhering to the plastic surface of culture plates or flasks (Fang and Eglen, 2017). Cells adhere to and grow on a flat surface, flattening morphologically and receiving nutrients and growth factors on one side during expansion (Neuhuber et al., 2008). This process is labor-intensive and susceptible to contamination due the open nature of the culture and to the number of cell passages required to generate sufficient cells for research purposes. Typically, 2D culture conditions are static and also lack monitoring *via* sensors and the ability to control culture conditions, which is undesirable for cell manufacturing (Martin et al., 2004). Primary MSC monolayer cultures can also become senescent and lose their phenotype following extensive passaging (Goepfert et al., 2010), which may impact on clinical efficacy (von Bahr et al., 2012). Thus, from a manufacturing perspective, given the relative rareness of MSCs in tissues and the quantity of cells required for clinical use, multiple master cell banks from multiple donors may have to be produced every year. This driver towards higher passage number and maximal expansion to derive the maximum number of patient doses from a single master cell bank needs to be balanced against potential reduced clinical efficacy. Further drawbacks of planar culture systems include the large surface areas required for cell growth at clinical scales, sizeable volumes of liquids to be manipulated during media changes, passages, and cell harvesting, and large incubators are required which occupy considerable space in clean rooms (Campbell et al., 2015; Merten, 2015).

To increase cell number under 2D conditions, the surface area of the culture dishes used is increased using multi-layered flasks, or cell stackers (Rowley et al., 2012). Small-, medium-, and large-scale cell manufacture in planar, 2D static culture are represented in **Figure 2** as tissue culture flasks through to 10-layer and 40-layer stacked systems. Several cell stackers are commercially available, including the Corning® CellSTACK and Nunc™ Cell Factory™. This manufacturing method is referred to as “scale-out” expansion, wherein the expansion unit size remains constant and parallel units are multiplied (**Figure 2**). However, this technique results in restricted surface-to-volume ratio, creating a bottleneck in the manufacturing process. The environment within cell stackers is also non-homogenous: each flask constitutes a different microenvironment that is susceptible to contamination, batch-to-batch variability and non-uniform surface treatment between suppliers (Jossen et al., 2018). Furthermore, manual handling and downstream cell processing constraints limit the potential of scale-out techniques. The high MSC doses required for therapeutic infusion [around 10^6^ cells per kg of patient (Jung et al., 2012)] necessitate “scale-up” methods.

Scale-up expansion refers to the increase in overall manufacturing scale that occurs in technologies such as bioreactors. A number of bioreactor types are depicted in **Figure 2**, including stirred tank, wave bag, and vertical wheel. In the microcarrier culturing system devised by van Wezel in 1967 (van Wezel, 1967), cells are propagated on the surface of microcarriers and expanded in suspension of growth medium *via* slow agitation. From this, stirred or mixed bioreactor systems incorporating microcarriers have been developed to provide densities of 10^6^ to 10^7^ cells/mL, becoming preferable to cell stackers for the generation of therapeutic cells (Fan et al., 2015). Furthermore, the shorter culture time bioreactor systems required to generate comparable cell numbers to tissue culture flasks can minimise the risk of MSC senescence and phenotypic changes due to culturing in serum (Mizukami et al., 2016). Other approaches used to increase the cell growth surface area, without increasing the footprint of the bioreactor include the use of hollow fibre bioreactors (Tozetti et al., 2017; Savelli et al., 2018) as well as fixed bed perfusion systems (Sart et al., 2014). An important feature of many scale-out systems is the ability to be able to operate them in a functionally closed manner. This means that the bioreactor can be opened to make a connection and then returned to the closed state. In this way, the contents of the bioreactor are not exposed to the room environment. This presents a distinct advantage since a number of units can operate in the same room without physical separation from each other.

### Stirred-Tank and Other Dynamic Bioreactors

Typical stirred-tank bioreactors are usually cylindrical vessels with an impeller providing constant movement and are the most widely used scaled up bioreactor system used for MSC-based cell therapies, particularly allogeneic cell therapies where large cell numbers are required to be manufactured. The stirred tank configuration results in effective mixing, however, with non-homogeneous flows which can be turbulent in some conditions or regions within the bioreactor (Berry et al., 2016; Tsai and Ma, 2016). Bioreactor scale-up techniques facilitate dynamic suspension cultures which are very different to static 2D cultures. Cells within bioreactors can be expanded as suspended cell aggregates or seeded onto small solid spheres called microcarriers. For MSCs, expansion using this approach has generally been found to retain a stable phenotype (Caron et al., 2012) at least when only the minimum definition of an MSC is considered. As self-assembling cell aggregates or spheroids mimic *in-situ* conditions, cell morphology is more representative of that in bodily tissue(Edmondson et al., 2014). The medium in which the cells aggregate to form spheroids includes the need for adhesive molecules to facilitate cell-cell attachment, including laminins, integrins, E-cadherin, and vitronectin (Badenes et al., 2016). However, for GMP production, these recombinant human proteins are expensive, making viable large-scale manufacture difficult (Villa-Diaz et al., 2013).

Microcarrier-based culture systems are, in principle, particularly well-suited for MSC expansion. Microcarrier beads have a large surface area compared to 2D systems, maximizing MSC attachment. Bioreactors using microcarriers can also operate at higher densities, reducing supply costs, or cost of goods (COGs). For example, a study investigating the use of microcarrier-based MSC expansion of 2.5 L cultures in a stirred tank bioreactor system found that the larger volume cultures outperformed small 100-mL volume “spinner flask” cultures, producing cells with the phenotype, key morphology, and differentiation capacity that conformed to the ISCT definition of MSCs (Rafiq et al., 2013). Microcarriers are made from various materials and may be coated with biologically active proteins and peptides (e.g., vitronectin and fibronectin) (Melkoumian et al., 2010). Furthermore, microcarrier-based technology can be operated as a closed culture system and is compatible with sterilization procedures, which is essential when considering therapeutic applications (Schop et al., 2008).

Despite their advantages, three-dimensional (3D) scale-up manufacturing systems utilizing microcarriers and stirred tank systems raise potential issues. Further improvements tailored to the expansion of MSCs in dynamic culture systems are required to achieve unchanging and reproducible MSC production for biological research and eventual clinical application. In addition, research is still required to fully understand the link more broadly between manufacturing methodology and clinical efficacy and how to optimise manufacturing to achieve the best clinical outcomes. This is particularly relevant for MSCs as they are applied to a wide range of disease indications, which may require different properties which can be tailored on a disease basis using optimized manufacturing.

#### MSC Scale-Up in Stirred-Tank Bioreactor Systems

Bioreactor systems commonly used pose a number of possible issues for MSC scale-up production. This is largely because such systems were initially designed to carry out chemical reactions at scale and later adapted to cell culture in the form of bioprocessing or therapeutic protein production from non-adherent cells (e.g., CHO cells) (Nienow, 2006). For the manufacture of cell-based therapies, retention of cell function and quality is of principal importance, yet this aspect is often overlooked when adapting scale-up manufacture systems to large-scale production of MSCs.

A range of different commercial bioreactors are available for scale-up MSC manufacture (Badenes et al., 2016) (**Figure 2**). Bioreactor performance in supporting MSC growth and phenotypic maintenance cannot be the only variable considered when selecting a bioreactor. Criteria such as the ability to operate in a functionally closed way, simplicity of operation, disposability, sterility, single use, ability to incorporate online monitoring and control, automation, ease of harvest and time- and cost-effectiveness must also be taken into account (Caruso et al., 2014; Badenes et al., 2016). This must be balanced with practical considerations, such as low costs and the ability to achieve high cell densities.

Stirred-tank bioreactor systems can be readily operated and cell culture volumes can be scaled up with computer-controlled online monitoring equipment which control process variables such as pH, temperature, and dissolved oxygen and carbon dioxide concentrations (Tsai and Ma, 2016). However, stirred bioreactors also introduce an important complication: fluid mechanics (Odeleye et al., 2014; Berry et al., 2016). Cells in a bioreactor are constantly exposed to shear stress induced by mechanical agitation of impellers or wheels. MSCs are particularly sensitive to this stress, which can lead to cell damage, premature detachment from microcarriers, priming to a specific differentiation lineage or affect immunomodulatory properties (Stathopoulos and Hellums, 1985; Dos Santos et al., 2014; Das et al., 2019). These effects must be recognized and controlled for when expanding MSCs on microcarriers in a stirred bioreactor system. Ultimately, a dynamic culture system utilizing microcarriers is complex and presents different challenges to 2D systems. Aggregation of microcarriers is of particular relevance as their presence may reduce cell harvest efficiency. An approach taken to minimise aggregation is to periodically add more microcarriers, increasing the culture surface and allowing cells to migrate from confluent microcarriers to sparely populated or empty microcarriers (Ferrari et al., 2012; Rafiq et al., 2018). From a feasibility point of view expansion of bone marrow derived MSCs has been carried out in single use stirred tank bioreactors at 3 and 50 L (Lawson et al., 2017). Expansion in HPL supplemented media was enhanced compared to FBS and a 43-fold expansion was obtained in 11 days at a 50 L culture volume scale. Maintenance of MSC phenotype according to the ISCT definition was maintained as well as immunosuppressive properties.

As MSCs are anchorage-dependent, they must be easily separated from the substrate on which they are cultured without changing their immunophenotype, secretome or differentiation capacity, all of which are strongly related to clinical efficacy. Cell harvesting in dynamic systems is often conducted with a proteolytic enzyme such as trypsin (alone or in combination with chelating agents such as EDTA) to separate cells from microcarriers and cell-microcarrier aggregates, followed by filtering through an appropriate mesh to remove the microcarriers and large aggregates (Lindskog et al., 1987). Unlike monolayer cell culturing strategies, microcarrier–MSC complexes require especially complex disassociation methods and detachment efficiencies tend to vary. Several studies have treated cell-microcarrier complexes with trypsin at high concentrations or for long periods of time (Frauenschuh et al., 2007; Schop et al., 2008; Dos Santos et al., 2014). This treatment is known to cause MSC damage or induce phenotypic changes. For example, MSCs treated with 0.25% trypsin-EDTA solution for 5, 30, and 90 min at room temperature demonstrated decreased CD105 expression with time (Potapova et al., 2008). Other studies have investigated alternative proteolytic enzymes, such as collagenase and dispase, to harvest MSCs by digesting macroporous microcarriers. This approach limits cell damage and increases detachment numbers (Rubin et al., 2007; Sart et al., 2009). However, certain cell surface molecules have also been shown to be downregulated or cleaved upon cell treatment with these enzymes (Autengruber et al., 2012; Taghizadeh et al., 2018).

Alternatively, the use of thermosensitive microcarriers, which detached MSC-microcarrier complexes by decreasing the culture temperature, showed that cell detachment *via* temperature change reduced MSC apoptosis and cell death during harvesting, suggesting that thermosensitive microcarriers are effective in MSC culturing (Yang et al., 2010). There are a number of potential issues for thermosensitive microcarriers, including cell aggregates which may also need enzymatic digestion. In any case, it is crucial to consider the cell type and microcarrier type and identify an optimal enzymatic protocol to maximise the quantity and quality of cells harvested.

Stirred-tank bioreactors offer a promising approach for generating sufficient cell numbers under controlled scale-up conditions. However, they are not tailored to or optimized for MSC expansion. Considerations must be made towards maintaining batch-to-batch standardization, cell yields, and cytokine and growth factor secretions for industrial and clinical translation. The effects of microcarrier culture systems on the MSC secretome must be taken into consideration, as the secretome is considered an integral indication of therapeutic functionality. An outstanding question is whether the MSC secretome is changed in dynamic by scale-up manufacturing systems from that obtained in 2D culture systems. A newer technology, that of a vertical wheel bioreactor (see **Figure 2**) which is scalable to 500 L culture volumes, has been evaluated in HPL-supplemented media for umbilical cord-derived MSCs (UC-MSCs) and A-MSCs and an economic evaluation against static 2D culture carried out (de Sousa Pinto et al., 2019). It was found that significant cost reductions could be obtained (up to 50% in some cases) using this type of bioreactors system and microcarriers. Another advantage of using a vertical wheel instead of an impeller for mixing is that of reduced shear stress (Sousa et al., 2015), as the impact of shear stress on cell phenotype, differentiation capacity and secretome is largely unknown.

## Microcarriers

Microcarriers are small, spherical beads which allow production of cells at a high culture density due to the much larger culture surface area to media volume ratio. Stirring in the bioreactor maintains the microcarriers in suspension in a bioreactor (Caruso et al., 2014). They were traditionally employed to culture primary cells and anchorage-dependent cell lines for vaccine production, pharmaceutical production, and cell population expansion (Nilsson, 1988). Commercially available microcarriers are engineered for specific applications and vary in chemical composition, charge, surface coatings, and porosity (Malda and Frondoza, 2006) and allow cells to be cultured at a higher surface area per media volume than in planar culture.

Microcarriers are composed of various materials including polystyrene, dextran, and glass. Their surface can be functionalized with in different ways (e.g., *via* a coating) to maximize cell attachment and cell culture performance. This is largely accomplished by chemically derivatizing the microcarrier surface with functional groups, such as positively or negatively charged groups, biological materials (e.g., gelatine, collagen, fibronectin) or other small molecules such as peptides (Badenes et al., 2016). Unless chemically modified with a positively charged group, synthetic microcarriers (e.g., glass, dextran, and polystyrene) are generally negatively charged.

### MSC Attachment to Microcarriers

**Figure 3:**
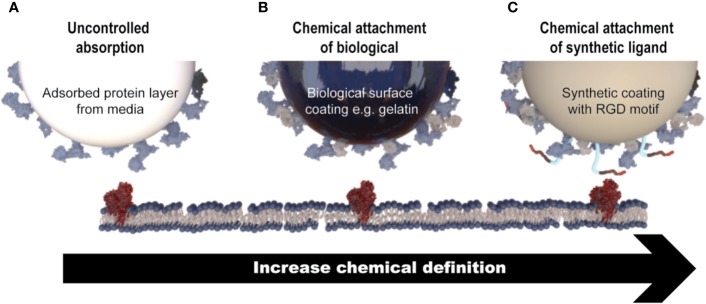
Mechanisms of MSC attachment to microcarriers. **(A)** Cell attachment is facilitated through non-specific protein adsorption on the surface of microcarriers that do not contain a coating of any description (e.g., Solohill Plastic). **(B)** Microcarriers that contain a coating of a biologically derived molecule (e.g., gelatin) which facilitates cell attachment through native cell attachment motifs. **(C)** Microcarriers which contain a synthetic coating with a chemically synthesized cell attachment motif, for example a short peptide sequence (e.g., Synthemax^®^).

As MSC growth is anchorage-dependent, interactions between the microcarrier surface, cells, and surrounding medium are critical for the manufacture of healthy cells. The microcarrier surface is quickly “conditioned” by non-specific protein adsorption from media supplements, which facilitates cell attachment (Wang et al., 2012). Protein adsorption onto the microcarrier surface is driven largely by electrostatic, ionic or van der Waals forces, hydrophobic interactions, and hydrogen bonding interactions (Petry et al., 2016). Alternatively, microcarrier surfaces can be functionalized with biologically derived molecules (such as proteins or protein fragment), to which MSCs attach *via* adhesion motifs (Melkoumian et al., 2010). Alternatively, a synthetic coating containing chemically synthesized cell adhesion motifs, such as RGD peptides, can be chemically attached to the surface of the microcarriers (e.g., Synthmax microcarriers). These types of microcarriers, which are generally known as chemically defined, would generally be preferred from a regulatory point of view (**Figure 3**).

**Figure 4:**
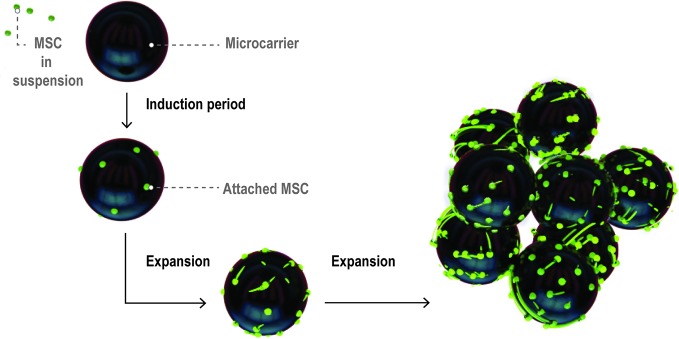
Visual representation of MSC expansion on microcarriers over time within a bioreactor. Figure adapted from (Caruso et al., 2014). MSCs initially attach at low coverage in a rounded morphology then flatten and spread over the induction period. The cells then enter a growth phase and expand to cover a large proportion of the microcarrier surface area.

As MSCs attach to microcarriers (known as the induction period of the culture), their phenotype changes from rounded to spread and fibroblastic (Battista et al., 2005) (**Figure 4**). Following the induction period, MSC expansion occurs. During cell expansion, the microcarrier growth surface interacts with cell surface integrins, the principal receptors mediating cell-matrix or cell-surface adhesion (Berrier and Yamada, 2007). Cell surface integrins are activated, adopt a heterodimer formation, and initiate signaling cascades which activate downstream gene expression and ultimately regulate cell morphology and behavior including attachment, spreading, proliferation, migration, and differentiation (Berrier and Yamada, 2007).

It is important to note that growth and harvesting of MSCs on microcarriers is different than in 2D microenvironments, as indicated in **Figure 4**. The surfaces are curved on the length scale of MSCs, which can wrap around the microcarrier surface and even bridge across microcarriers. There is a limited surface area per bead which means that cells on individual microcarriers will become confluent at different times, depending on how uniform the attachment density carries from bean to bean. Cells can transfer between beads or onto the surface of pristine beads added at different time points through the culture period (Derakhti et al., 2019). Aggregation of microcarriers through cell bridging is common and can impact ease of harvesting for cells trapped between beads.

The surface properties of commercial microcarriers can be tuned to facilitate this attachment process. For example, microcarrier surfaces are often coated with growth- and attachment-promoting proteins to encourage cell adhesion. Many media proteins can be used, including native or denatured collagen, fibronectin, laminin, and vitronectin (Melkoumian et al., 2010). Each protein is recognized by specific integrin heterodimers on the MSC surface (Plow et al., 2000; Docheva et al., 2007; Niehage et al., 2011) (**Table 2**). Integrin expression in MSCs differs by harvest tissue source: A-MSCs express the integrin subunits α_1_, α_2_, α_3_, α_5_, α_7_, α_8_, α_11_, α_v_, β_1_, β_3_, and β_5_ which bind *via* integrin receptors to their respective attachment proteins (De Ugarte et al., 2003; Goessler et al., 2008) (**Table 2**). In one study, actin organization was linked to more efficient expansion of MSCs on a range of microcarriers (Sart et al., 2013).

**Table 2:** MSCs express integrin heterodimers that attach to specific media proteins (Plow et al., 2000, Docheva et al., 2007, Niehage et al., 2011).

Attachment protein	MSC-expressed integrin subunits
Native collagen	α_1_β_1_, α_2_β_1_, α_11_β_1_, and α_Ib_β_3_
Denatured collagen	α_5_β_1_, α_v_β_3_, and α_IIb_β_3_
Fibronectin	α_2_β_1_, α_3_β_1_, α_4_β_1_, α_4_β_7_, α_5_β_1_, α_8_β_1_, α_v_β_1_, α_v_β_3_, α_v_β_5_, α_v_β_6_, α_v_β_8_, and α_IIb_β_3_
Laminin	α_1_β_1_, α_2_β_1_, α_6_β_1_, α_7_β_1_, α_6_β_4_, and α_v_β_3_
Vitronectin	α_v_β_1_, α_v_β_3_, α_v_β_5_, α_IIB_β_3_

Microcarrier matrix materials can also be selected from three categories: natural polymer, synthetic, and glass. Natural polymers, such as collagen, gelatine, dextran, and pectin, are commonly used as they are easily obtained, biocompatible, and inexpensive (Zhang et al., 2009; Zhou et al., 2011). Collagen- or gelatine-based microcarriers or coatings express attachment molecules to facilitate attachment (Geiger et al., 2001; Bertolo et al., 2015). Thus, they have the advantage of easy cell detachment, limiting cell damage. Furthermore, gelatine microcarriers are capable of enzymatic digestion, leaving only cells in suspension (Lönnqvist et al., 2015). However, biological materials can be problematic in the context of cell manufacture as regulatory agencies recommend the avoidance of mammal-derived materials to reduce the risk of contamination when MSC products are used in the clinic (Halme and Kessler, 2006; CHMP, 2013).

Therefore, cell manufacturers are increasingly focusing on synthetic polymer-based microcarriers which are largely composed of polystyrene (Rafiq et al., 2016). When the microcarrier surface does not contain biological attachment molecules, media attachment factors (particularly fibronectin or vitronectin) adsorb to the microcarrier surface and promote cell attachment and integrin binding (van Wachem et al., 1985; Evans et al., 2004). Alternatively, a chemically defined synthetic attachment substrate can be developed by coating the microcarrier with chemically synthesized materials that mimic the ligands of cell surface adhesive molecules. Thus, treatment allows synthetic microcarriers to facilitate cell adhesion and proliferation. Studies have indicated that various microcarrier matrix materials can support MSC growth, including polystyrene (Tseng et al., 2012), glass (Elseberg et al., 2012), decellularized adipose tissue (Turner and Flynn, 2012), gelatine (Eibes et al., 2010; Chen et al., 2011) and dextran (Hewitt et al., 2011).

### Commercially Available Microcarriers

A wide range of microcarriers are commercially available, enabling researchers to select one that suits their cell line, type, and purpose of cell expansion. Commercially available microcarriers can be categorized into six groups (**Table 3**). This section will discuss in further detail three popular microcarriers which have been in most cases widely used for MSC expansion: GE Healthcare's Cytodex®, Corning® Synthemax®, and SoloHill® Plastic. Selection of microcarriers is generally carried out be screening a range of microcarriers for attachment, growth, differentiation potential (Sart et al., 2013). Other parameters such as actin organization of attached MSCs may influence these outcomes as noted above. While this review is focussed on the use of commercially available microcarriers for scaled manufacturing of cells (Badenes et al., 2016; Chen et al., 2020), there are a number of other microcarrier systems in development which are used in other applications including tissue engineering (Shekaran et al., 2016; Lam et al., 2017; Zhou et al., 2020).

**Table 3:** Commercially available microcarriers (Chen et al,. 2013).

Microcarrier type	Non-porous/smooth	Collagen coated	ECM coated	Non-modified	Macroporous	Weighted
Example	Polystyrene microcarrier, e.g., plastic microporous microcarrier	Cytodex® 3	Pronectin-F	Glass beads, tissue culture polystyrene microcarriers	Cytopore, Cultispher®	Cytoline®
Properties	May incorporate a surface charge	Chemically coupled collagen	Coated with recombinant protein with a repeat RGD sequence	A high negative surface charge	Pore ranges in the range of 10–70 μm on microcarrier surface	Macroporous and the microcarrier matrix is made denser using silica
Suitable conditions	Enable culturing of adherent cells that form a continuous monolayer of cells on the surface of microcarriers in suspension	Enable culturing of sensitive cells with low plating efficiency, coating increases efficacy of cell harvest	Enable culturing of sensitive cells in serum-free conditions	Enable culturing of any anchorage dependent cell line in suspension	Provide higher surface areas for growth and offer better mechanical protection to cells from shear stress	Enable culturing in fluidized bed perfusion cultures

#### Dextran Beads: Cytodex® 1 and Cytodex® 3

Produced by GE Healthcare, Cytodex® 1 and 3 are dextran beads. Cytodex® 1 is positively charged while Cytodex® 3 features a denatured collagen coating. These biologic properties lead to positive results when culturing MSCs (Chen et al., 2013).

As MSCs express multiple integrin subunits that facilitate attachment to denatured collagen, microcarrier-MSC attachment on Cytodex® 3 is expected to be high. This is consistent with results from Goh et al. (2013), who achieved a 12- to 16-fold expansion efficiency (6×10^5^–8×10^5^ cells/mL) of human fetal MSCs on Cytodex® 3 microcarriers, compared to 4- to 6-fold expansion using traditional monolayer culture (1.2×10^5^–1.8×10^5^ cells/mL). The human fetal MSCs maintained colony-forming capacity, doubling times, and immunophenotype post-Cytodex® 3 expansion. Similarly, Frauenschuh and colleagues found that MSCs had greater than 80% attachment on Cytodex® 1 microcarriers following three hours of incubation (Frauenschuh et al., 2007). However, recent research by Lin et al. established that similar levels of cell adhesion, growth, and differentiation outcomes were achieved on Cytodex® 1 and 3 microcarriers (Lin et al., 2017). Thus, it may be concluded that microcarrier size, matrices, and surface nature are unlikely to be as crucial in determining MSC yield and differentiation outcomes as might be expected. Despite previous successes using Cytodex® microcarriers, their use is limited in a therapeutic context as these microcarriers are not xeno-free, leading to a risk of contamination through the introduction of adventitious xenogeneic agents (Felka et al., 2010).

#### Synthetic Peptide Surface Microcarrier: Synthemax®

The xeno-free Corning® Synthemax® Surface features a short peptide sequence derived from the vitronectin protein to mimic the biological ligand for cell adhesion (Melkoumian et al., 2010). The peptide is based on the Arg-Gly-Asp (RGD) motif and immobilized on an acrylate coating. Synthemax^®^ microcarriers may be obtained already coated in two different peptide surface densities or the Synthemax® surface can be added to synthetic or biological microcarriers through an adsorption process to support MSC attachment and growth.

Previous research has found that the Synthemax® Surface can replace ECM proteins to facilitate efficient MSC attachment, support the long-term culture of BM-MSCs and maintain cell surface antigen expression profile following expansion (Dolley-Sonneville et al., 2013). The study calculated cell yield to be significantly higher compared to traditional BM-MSC culture in serum-containing medium. A similar study demonstrated that the Synthemax® Surface peptide recapitulates integrin-ECM engagement of human embryonic stem cell (hESC) comparable to those grown on Matrigel-coated substrates (Jin et al., 2012). The synthetic ligand interacted with human induced pluripotent stem cells (hIPSCs) *via* the integrin α_v_β_5_ units, demonstrating its comparability to vitronectin. Lambshead *et al*. observed human pluripotent stem cells (hPSCs) cultured on Synthemax® coated plates and flasks were morphologically indistinguishable from those cultured in control flasks coated with Geltrex (Lambshead et al., 2018). Accordingly, the genetic stability and pluripotency of hPSCs was maintained on Synthemax® surface as assessed by the PluriTest™ assay (Muller et al., 2011).

Findings regarding cell yield are consistent with other reports in the literature regarding the performance of the Synthemax® Surface (Meng et al., 2010). Importantly, the Synthemax^®^ Surface is xeno-free and therefore compatible with serum-free media. This eliminates the risk of xeno-contamination inherent in the use of animal-derived products, a strong advantage as compared to Plastic, Plastic Plus and Star-Plus microcarriers in a therapeutic context. However, its use may be limited by financial considerations: the cost of goods may be higher for microcarriers with synthetic coatings than for uncoated styrene microcarriers.

#### Cross-Linked Polystyrene Microcarriers: Plastic, Plastic Plus, Star-Plus

The SoloHill® range of styrene copolymer microcarriers have no specialized coating and may incorporate a surface charge to enhance protein adsorption from media supplements which facilitates MSC and attachment at an acceptable level. In the case of the Plastic microcarriers, the surface of the particles is modified to make them more hydrophilic than the base polystyrene material and is most likely negatively charged. Attachment of MSCs to Plastic is facilitated by the adsorption of extracellular matrix (ECM) proteins present in the media (Dolley-Sonneville et al., 2013). Relatively little is known about the proteins adsorbed from culture media onto microcarrier growth surfaces. The adsorbed layer on SoloHill® microcarriers are likely a complex mixture of partially denatured proteins which is highly difficult to characterise (Wang et al., 2012).

Cells derived from vertebrates (such as MSCs) carry a heterogeneous negative surface charge (Varki and Gagneux, 2012). During the cell-growth surface adhesion process, electrostatic forces and van der Waals forces play an important role in the interaction of the cell and growth surface (the microcarrier plus adsorbed protein layer from the media) (Petry et al., 2016). Initially positive surfaces (e.g., Plastic Plus, Star-Plus) become less positively charged over time as more proteins are attracted to and adsorb to its surface, changing the overall net charge to negative. Plastic, which is not chemically modified to incorporate a positive charge, is negatively charged. Relatively hydrophobic surfaces such as the SoloHill® microcarriers may attract the types of proteins that facilitate MSC attachment (Grinnell and Feld, 1981). The initial surface sign, magnitude of charge, and degree of hydrophobicity are determinants for the types, quantity, and nature of adsorbed proteins on the surface of microcarriers. Microcarrier properties which are conducive to MSC attachment and growth are generally discovered by screening a range of microcarriers, often in small volume, static cultures (Rafiq et al., 2016).

It is proposed that uncoated microcarriers with positive (e.g., Plastic Plus, Star-Plus) or negative (e.g., Plastic) charge will demonstrate better cell-surface attachment due to their ability to encourage protein adsorption from the media onto their surfaces which facilitates MSC attachment and growth. In a previous study, a greater yield of UC-MSCs was obtained on Plastic and Plastic Plus microcarriers compared to Pronectin-F (an RGD polymer-coated microcarrier) and glass microcarriers (Petry et al., 2016). A slightly higher cell yield was obtained on Plastic Plus microcarriers compared to Plastic. This establishes the preference of UC-MSCs for polymer substrates over glass. Furthermore, Rafiq and colleagues selected Plastic microcarriers as optimal for BM-MSC expansion following a systematic evaluation of 13 microcarriers (Rafiq et al., 2016). BM-MSC immunophenotype and differentiation capacity was unchanged following harvesting on polystyrene microcarriers.

In comparison to the well-characterized abilities of Plastic and Plastic Plus, Star-Plus is a relatively new microcarrier and extensive research on its relative usefulness in MSC scale-up expansions has not yet been conducted. All plastic microcarriers discussed here are xeno-free and, therefore, pose no risk for contamination of cells for therapeutic purposes. However, a significant disadvantage of these types of microcarriers is that they cannot be readily used in serum-free or chemically defined synthetic media as these do not contain serum proteins typically. Thus, a pre-conditioning step with recombinantly produced, GMP-grade human ECM proteins may be required, increasing costs and process complexity.

### Microcarriers and MSC Fate

The effects of substrate stiffness on MSC properties must be considered, MSCs specify cell lineage with respect to tissue-level elasticity (Engler et al., 2006). The spectrum of stiff to soft substrates can alter MSC surface markers, with MSCs lineage markers primed to neurogenic following growth on low-stiffness substrates, myogenic on medium-stiffness substrates and osteogenic on stiff substrates. Although the effect of MSC substrate stiffness on cell differentiation pathways are well known, there is a gap in the literature regarding substrate effect on MSC secretome, and thus immunomodulation. Furthermore, studies focussing on MSC expansion on microcarriers have not elucidated the effects, if any, of microcarrier stiffness on the MSC secretome.

The attachment of microcarriers to MSCs *via* ligand-receptor complexes has been shown to transmit physiochemical signals within the cell *via* mechanotransduction mechanisms, thereby altering cell fate (Nomizu et al., 1995). ECM proteins from cell culture supplements (or derivative motifs found on the surface of microcarriers) bind to specific MSC cell surface integrin receptors, which activate intracellular signaling pathways and controls gene expression, cytoskeletal organization, and cell morphology (Nomizu et al., 1995). Each integrin receptor can bind to a multitude of ECM proteins and stimulate at least six different classes of intracellular signaling molecules: protein tyrosine kinases, serine/threonine kinases, lipid kinases, lipid phosphates, protein phosphatases, and intracellular ion fluxes (Schwartz and Ginsberg, 2002). Through differential attachment, different microcarriers may alter MSC immunophenotype, differentiation capacity, and possibly secretome (**Figure 4**).

A study by Salasznyk and colleagues determined that culturing hMSCs on vitronectin and collagen I substrates can promote their osteogenic differentiation *via* ECM contact, inducing differentiation (Salasznyk et al., 2004). These findings have been expanded by the demonstration that MSCs propagated and harvested from microcarriers demonstrate higher osteogenic potency than those cultured in traditional monolayer cultures (Goh et al., 2013). Their results suggest that MSC culture on microcarriers resulted in a change in cell phenotype, perhaps caused by the activation of different intracellular signaling molecules following attachment. There is a body of evidence that suggests mechanical properties may prime MSCs for particular differentiation pathways, and potentially alter gene expression (Frith et al., 2010; Frith et al., 2012a; Frith et al., 2012b; Kusuma et al., 2017; Lin et al., 2017; Frith et al., 2018). This raises the question of whether the mode of MSC growth in the expansion phase affects other aspects of MSC immunophenotype, such as their secretome.

Teixeira and colleagues considered modulating MSC secretome by changing the culture environment and concluded that dynamic culture conditions may be a strong asset in regenerative strategies revolving around the use of the MSC secretome (Teixeira et al., 2016). Although the study focussed on computer-controlled bioreactors, the findings can be expanded to MSCs cultured on a range of microcarriers. A recent novel study investigated the role of microenvironment surface structure on cytokine secretion profile (Leuning et al., 2018). The group cultured BM-MSCs and kidney perivascular stromal cells (kPSCs) on unique topographies and measured any changes in cytokine and growth factor secretion compared to the same cells grown in planar culture. Although functionally different, both BM-MSCs and kPSCs displayed different cell morphologies and cytokine secretion profiles when grown on varying topographies. Their findings support the hypothesis that MSC secretome is influenced by microenvironment structure such as focal adhesion density, size, and protein recruitment. Thus, MSC immunomodulatory function may be capable of manipulation in an engineered setting (such as microcarrier expansion). The implication that microcarrier surface topography in bioreactor expansion should be taken into account to preserve therapeutic properties of MSCs should be examined in further detail.

Apart from the study by Leuning *et al*., research where screening of microcarriers is carried out for the purposes of selecting the best microcarrier for growth of MSCs has not considered changes in cell secretome, other than testing the cells produced in simple, immunosuppression tests. This may not be predictive for how the cells will behave *in vivo*. Furthermore, a relationship between the expansion surface (such as microcarriers) and MSC cell contact-dependent immunosuppression has not been investigated thoroughly in the prior literature. Thus, a microcarrier best suited for the desired MSC secretome for clinical application has not been identified in previous research, which is remiss in the field as the therapeutic benefits of MSCs are often attributed to their secretome. Any changes in cell contact-induced immunosuppression or secretome may affect MSC immunomodulatory potential, which must be studied in detail prior to the licencing of therapeutics.

## Conclusions

MSCs exert immunomodulatory effects on innate and adaptive immune cells. They induce their effects through cell-to-cell contact and the release of cytokines and other bioactive molecules (Di Nicola et al., 2002). Research involving MSCs is intensifying due to their therapeutic potential for a variety of diseases, largely mediated by their immunosuppressive properties.

The large number of cells required for therapeutic infusions requires 3D scale-up technologies such as stirred-tank bioreactors. These technologies have advantages and disadvantages which are thoroughly researched in the literature. Microcarriers, on which MSCs are propagated in bioreactors, have a high surface area allowing high rates of attachment (Caruso et al., 2014). They can be chemically modified to further increase MSC attachment. Bioreactors themselves can be monitored by online sensors, allowing cell microenvironment variables to be maintained in tight parameters (Badenes et al., 2016). However, culturing in bioreactors presents issues such as shear stress on cells, inconsistent temperature and pH, and removal from microcarriers which may change MSC phenotype (Stathopoulos and Hellums, 1985; Lindskog et al., 1987; Dos Santos et al., 2014).

MSCs are known to actively respond to their culture microenvironment, including substrates they are propagated on, by secreting various cytokines and growth factors. These soluble factors are important constituents of the MSC secretome that underlie many of their immunomodulatory properties. However, scale-up manufacturing methods are not currently tailored for MSC expansion, and there is a lack of knowledge about whether MSC expansion on microcarriers alters the secretome and cell function. The establishment of a 3D MSC culture method that does not compromise the immunomodulatory properties of MSCs would drastically improve clinical feasibility. Advances in this area will need to take into account recent findings that challenge the tenet that MSCs need to remain viable for therapeutic efficacy.

In addition to the manufacturing considerations, the extensive efforts toward understanding MSC biology, their secretome, fate upon administration and interactions with a range of immune cells, and soluble factors need to intensify in order to delineate pathways through which MSC-mediated immunosuppression takes place. This will provide substantial foundation and direction to the engineering and pharmaceutical groups whose efforts in developing a commercial MSC product currently are blindsided by the lack of knowledge and immense speculation regarding MSC application. Shedding light in these aspects will almost certainly ensure a more translatable MSC product for tissue regeneration.

## Author Contributions

DC, TB, LM, and TH contributed conception and design of the manuscript. DC wrote the first draft of the manuscript. TB, LM, and TH wrote sections of the manuscript. All authors contributed to manuscript revision, read, and approved the submitted version.

## Funding

TH is supported by an R.D. Wright Career Development Fellowship (APP1107188) and a Project grant (APP1162499) from the National Health and Medical Research Council of Australia. The authors have received funding from Regeneus Limited outside of this work. The funder was not involved in any part of this research.

## Conflict of Interest

The authors declare that the research was conducted in the absence of any commercial or financial relationships that could be construed as a potential conflict of interest.

The reviewer CH declared a shared affiliation, with no collaboration, with the authors to the handling editor at the time of the review.
